# Supplementary material for: Aspergillus Sensitization and Allergic Bronchopulmonary Aspergillosis in Asthmatic Children: A Systematic Review and Meta-Analysis
Source: Diagnostics (Basel). 2023 Mar 1;13(5):922. doi: 10.3390/diagnostics13050922 (PMC10001349; doi:10.3390/diagnostics13050922)

Table S1: Newcastle Ottawa score to assess the quality of the studies included in the systematic review of literature and meta-analysis

**Reference**	**Selection (max 5)**							**Overall score**
**Study**	**Representativeness (1)**	**Sample size (1)**	**Non-respondents (1)**	**Ascertaining exposure (2)**	**Comparability (2)**	**Outcome Assessment (2)**	**Statistical test (1)**	
Hoehne JH, et al. (1973)[39]		*	*	**	*	**	*	8
Chetty A, et al. (1985)[40]	*		*	**	**	**	*	9
Koivikko A, et al. (1991)[41]	*	*	*	**	*	**	*	9
Korhonen K, et al. (2006)[42]	*	*	*	**	*	**	*	9
Knutsen AP, et al. (2010)[43]	*	*	*	**	*	**	*	9
Nabavi M, et al. (2010)[44]	*	*	*	**	*	**	*	9
Arroyave WD, et al. (2013)[45]	*	*	*	**	**	**	*	10
Vicencio AG, et al. (2014)[46]	*	*	*	**	*	**	*	9
Singh M, et al. (2015)[47]		*	*	**	*	**	*	8
AlKhater SA, et al. (2017)[48]	*	*	*	*	*	**	*	8
Byeon JH, et al. (2017)[49]	*	*	*	**	*	**	*	9
Lehmann S, et al. (2017)[50]	*	*	*	**	*	**	*	9
Gupta A, et al. (2018)[51]	*	*	*	*	*	**	*	8
Ishak SR, et al. (2020)[52]	*		*	**	*	**	*	8
Kumari J, et al. (2020)[53]		*	*	**	**	**	*	9
Welsh KG, et al. (2021)[9]	*	*	*	*	*	**	*	8

*Indicates the score assigned. A maximum of five, two, and three are possible for the ‘selection’, ‘comparability’ and the ‘outcome’ domains, respectively.
- indicates no score assigned

Figure S1: Funnel plot for the outcome of allergic bronchopulmonary aspergillosis in asthmatic children

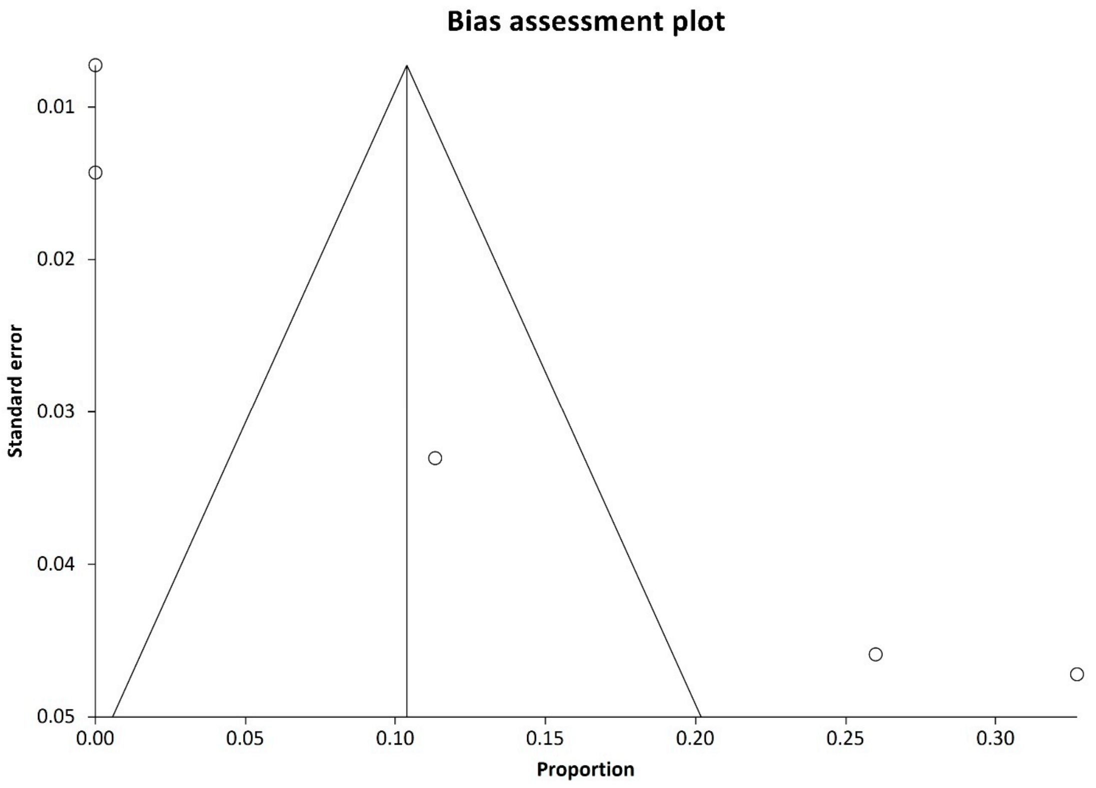

Supplement: Supplementary file 1 [file diagnostics-13-00922-s001.zip › diagnostics-2178465-supplementary.pdf]
